# Metagenomic landscape of peripancreatic necrotic pus in acute pancreatitis: pathogen ecology, resistance gene profiles, and disease-associated microbial patterns

**DOI:** 10.3389/fmicb.2026.1950013

**Published:** 2026-09-10

**Authors:** Haichao Li, Zhenzhen Shi, Ruoxi Zhang, Jinwei Yao, Jia Li, Dachuan Liu

**Affiliations:** 1 Department of General Surgery, Xuanwu Hospital, Capital Medical University, Beijing, China; 2 Department of Medicine, Dinfectome Inc., Nanjing, Jiangsu, China

**Keywords:** acute pancreatitis, antimicrobial resistance gene, metagenomic next-generation sequencing, peripancreatic necrotic pus, persistent organ failure

## Abstract

**Objective:**

To characterize the metagenomic landscape of intra-abdominal pus from surgical ICU patients using metagenomic next-generation sequencing (mNGS), compare pathogen ecology and resistance gene profiles between acute pancreatitis (AP)-associated and non-pancreatitis abdominal infection, and examine the association between pancreatitis-associated microbial features and persistent organ failure status.

**Methods:**

A total of 115 patients undergoing abdominal paracentesis and drainage in a surgical ICU were stratified into three groups: the POF group (AP with persistent organ failure, *n* = 35), the Non-POF group (AP without persistent organ failure, *n* = 49), and the Non-AP group (non-pancreatitis intra-abdominal infection, *n* = 31). Pus samples were analyzed by mNGS to characterize pathogen profiles, antimicrobial resistance gene (ARG) carriage, and microbial community structure.

**Results:**

Compared with Non-AP infections, both AP groups showed a convergent microbial profile dominated by ICU-associated pathogens, including *Enterococcus faecium*, *Klebsiella pneumoniae*, *Acinetobacter baumannii* complex, and *Pseudomonas aeruginosa*, with broad ARG detection profiles. ARG positivity was higher in AP patients than Non-AP controls (69.0% vs. 45.2%; *P* = 0.029), with frequent detection of Beta-lactam resistance genes, including carbapenemase-related genes. At sampling, alpha and beta diversity did not differ between POF and Non-POF groups, whereas both AP groups were clearly separated from Non-AP infections (*P* < 0.01). Among species enriched in Non-AP pus, *Enterococcus faecalis* remained inversely associated with AP status after adjustment for comorbidity burden, disease severity, antibiotic exposure, and ICU exposure (all *P* ≤ 0.006). The family-level random forest model achieved exploratory discriminatory performance for AP identification (AUC = 0.843).

**Conclusion:**

AP-associated peripancreatic pus harbored a distinct microbial ecological profile characterized by reduced community diversity, predominance of ICU-associated pathogens, and broad ARG detection profiles. This pattern was associated with AP status but not with POF at sampling. These findings may complement antimicrobial risk assessment and empirical treatment decisions when interpreted alongside culture-based susceptibility testing and clinical factors, but require validation in prospective multicenter studies.

## 1 Introduction

Acute pancreatitis (AP) is one of the leading causes of admission to emergency surgical ICUs. In 2021, the global age-standardized incidence reached 32.8 per 100,000 population (95% UI: 28.9–37.4), with incidence among adults aged 15–25 years trending upward over the same period (Liu B. et al., 2025). While the majority of episodes are self-limiting, roughly 20% progress to severe disease marked by persistent organ failure (POF) and pancreatic necrosis, with reported mortality ranging from 20 to 40% (Liţã Cofaru et al., 2025; Metri et al., 2024; Cai et al., 2025). Secondary infection of necrotic pancreatic tissue, termed infected pancreatic necrosis (IPN), is the complication most strongly linked to progressive disease and mortality (Ali et al., 2026). Prompt and accurate pathogen characterization is therefore critical once infection is confirmed or suspected.

Reliable microbiological diagnosis of IPN remains difficult in routine clinical practice (Baron et al., 2020). Conventional culture—still the reference standard—requires 48–72 h for results and yields false negatives in a substantial proportion of polymicrobial specimens (Rodriguez et al., 2008); when antibiotic decisions cannot be deferred, this delay carries direct clinical consequence. Metagenomic next-generation sequencing (mNGS) circumvents these constraints by enabling culture-independent, simultaneous detection of bacteria, fungi, viruses, and parasites from a single sample within approximately 24 h (Liu Y. et al., 2025). Most published mNGS studies in AP, however, have used blood or fine-needle aspirate as the source material (Baron et al., 2020; Hong et al., 2024); direct sequencing of peripancreatic necrotic pus—the sample closest to the site of infection—remains relatively rare, and the microbial composition of this compartment has not been systematically described.

The pathophysiology of IPN implicates the gut as a key source of infecting organisms. Disruption of the intestinal mucosal barrier and translocation of enteric bacteria to peripancreatic necrotic tissue are well-documented features of severe AP (Cui et al., 2025; Zhang et al., 2025), and the microbial community that colonizes necrotic tissue is shaped not only by the patient’s endogenous flora but also by the selective pressures of prolonged ICU stay and broad-spectrum antibiotic exposure. Prior work has consistently identified *Escherichia coli*, *Klebsiella pneumoniae*, and *Pseudomonas aeruginosa* as frequent and clinically consequential pathogens in IPN (Abulfaraj, 2025; Hong et al., 2024; Pauw et al., 2026), yet conventional methods cannot characterize the full ecological context in which these organisms exist—their relative dominance within the microbial community, co-occurring taxa, or resistance gene burden across the population. Whether the pus microbiota of AP patients differs compositionally from that of other intra-abdominal infections, and whether this composition is influenced by the degree of organ dysfunction, has not been examined through mNGS-based community profiling.

To address these questions, we collected peripancreatic necrotic pus from AP patients undergoing drainage in a surgical ICU and compared its microbial composition with that of pus from patients with non-pancreatitis intra-abdominal infection. Using mNGS, we characterized pathogen detection profiles, antimicrobial resistance gene (ARG) carriage, and overall community structure across groups stratified by clinical diagnosis and organ function status. Our primary aim was to determine whether AP-associated pus exhibits a distinct microbial pattern compared with other abdominal infections and whether this pattern differs according to organ function status, with the broader goal of providing microbiological insights relevant to antimicrobial risk assessment and empirical treatment considerations in critically ill patients with suspected IPN.

## 2 Materials and methods

### 2.1 Study population and clinical baseline data collection

**FIGURE 1:**
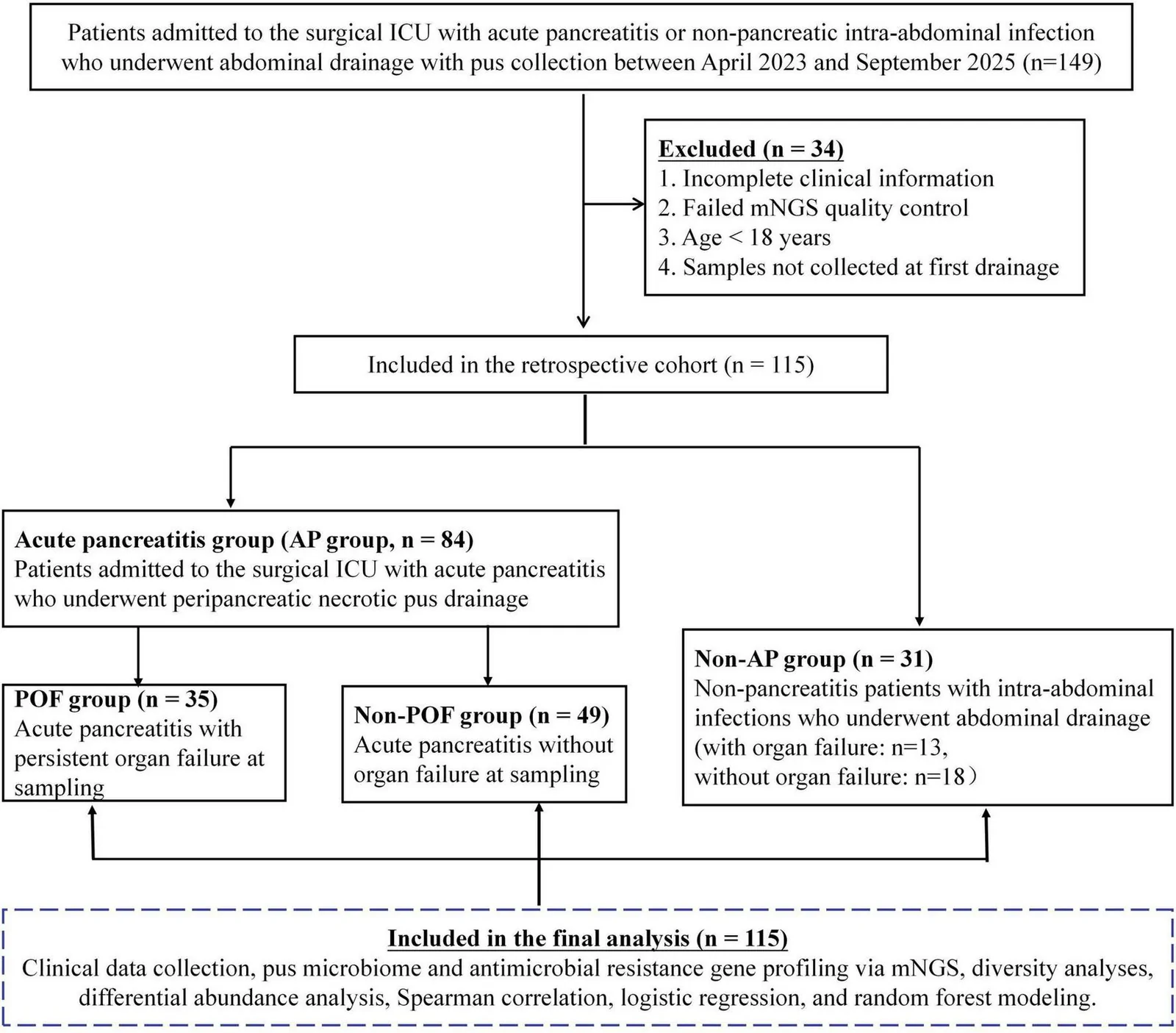
Study enrollment flowchart.

This single-center retrospective study enrolled consecutive patients admitted to the surgical ICU between April 2023 and September 2025 who underwent abdominal drainage with pus collection (Figure 1). The diagnosis of both AP-associated and Non-AP intra-abdominal infections was based on the final clinical diagnosis, established by two independent clinicians through comprehensive evaluation. AP patients were eligible if they had a confirmed clinical and radiological diagnosis of acute pancreatitis according to the International Association of Pancreatology Revised Guidelines on Acute Pancreatitis 2025 (IAP/APA/EPC/IPC/JPS Working Group, 2025), requiring at least two of the following: (1) characteristic acute-onset abdominal pain; (2) serum amylase or lipase ≥ 3 times the upper limit of normal; (3) characteristic findings on cross-sectional abdominal imaging (CT or MRI); and underwent drainage of peripancreatic necrotic pus. The Non-AP group was prespecified as a clinically relevant comparator comprising patients with non-pancreatic intra-abdominal infection, rather than a single etiological disease entity, the diagnosis of intra-abdominal infection was confirmed primarily by the acquisition of purulent material at abdominal drainage following ICU admission—a mandatory inclusion criterion for all Non-AP patients—supported by one or more of the following: clinical features (fever, abdominal pain, and peritoneal signs), laboratory findings indicative of infection (leukocytosis and/or elevated procalcitonin), and imaging evidence on abdominal CT (intra-abdominal fluid collection, abscess formation, or free gas). Patients were excluded for any of the following: incomplete clinical data, age under 18 years, missing or quality control-failed mNGS data, or samples not collected at the time of first drainage.

Enrolled patients were stratified into three groups according to clinical diagnosis and organ function status at the time of pus collection: the POF group (AP with persistent organ failure, defined as a modified Marshall score ≥ 2 in any organ system persisting for > 48 h at sampling; *n* = 35), the Non-POF group (AP with intact organ function at sampling; *n* = 49), and the Non-AP group (*n* = 31). Demographic characteristics, laboratory parameters, clinical manifestations, culture and antimicrobial susceptibility testing (AST) results, disease severity scores (APACHE II score and Charlson Comorbidity Index), ICU admission-to-sampling interval, pus collection modality, and pre-sampling antibiotic duration and class were all retrospectively retrieved from the electronic medical record system. The study was approved by the institutional ethics committee (approval ID: LYS[2025]210-001).

### 2.2 mNGS detection and pathogen and resistance gene analysis

Pus samples were collected at the time of each patient’s first abdominal paracentesis and drainage procedure. Depending on the location and accessibility of the collection and the clinical treatment strategy, pus specimens were obtained by CT/ultrasound-guided percutaneous drainage, endoscopic ultrasound-guided transmural drainage (EUS-TD), or surgical drainage. A 5 mL was transferred to a sterile container and transported on ice for storage. Genomic DNA was extracted using the TIANamp Magnetic DNA Kit (Tiangen, China) per the manufacturer’s protocol. Library preparation was performed with the Hieff NGS C130P2 OnePot II DNA Library Prep Kit (Yeasen Biotechnology, Shanghai), quality was assessed on an Agilent 2100 Bioanalyzer, and sequencing was carried out on the DIFSEQ-200 platform (Dinfectome, Nanjing) using 50-bp single-end reads, targeting approximately 20 million reads per sample.

Raw reads were demultiplexed with bcl2fastq2 (v2.20). Low-quality reads, adapter sequences, duplicates, and short fragments ( < 36 bp) were removed using Trimmomatic (v0.36). Human-derived reads were filtered by alignment to the human reference genome (hs37d5) with Bowtie2 (v2.2.6); non-human reads were retained for taxonomic classification with Kraken2 (v2.0.7) and species-level abundance estimation with Bracken (v2.5.0). The reference database comprised bacterial, fungal, viral, and parasitic genomes and scaffolds downloaded from GenBank release 238.^1^

A species was considered mNGS-positive if: (1) at least one species-specific read was detected for *Mycobacterium*, *Nocardia*, or *Legionella pneumophila*; (2) at least three non-overlapping reads were detected for all other bacteria, fungi, viruses, and parasites; or (3) the read count exceeded that of the negative no-template control (NTC) by at least 10-fold when the same species was detected in the NTC.

ARG profiling was performed by aligning non-human reads against the Comprehensive Antibiotic Resistance Database (CARD) using DIAMOND in blastx mode. Candidate ARG hits were filtered using predefined alignment-quality criteria, including e-value, sequence identity, alignment coverage, and read support, to minimize false-positive detections. Detected ARGs passing these quality-control criteria were subsequently normalized by sequencing depth for abundance estimation. ARGs were assigned to ten categories following CARD nomenclature: Beta-lactam, Aminoglycoside, Tetracycline, Glycopeptide, Sulfonamide, Trimethoprim, Fluoroquinolone, Macrolide/Lincosamide, Phenicol, and Oxazolidinone. Multiple ARGs within the same category in a single sample were counted as one positive event; categories were ranked by mean detection rate across the three groups. The co-occurrence rate between an ARG category and a microbial species was calculated as the number of samples positive for both divided by the number of samples positive for that species, expressed as a percentage; eight clinically relevant species were included using all samples pooled, and ARG category columns were clustered by Ward’s method with Euclidean distance. Pathogen and ARG detection rates were compared between the combined AP group (POF + Non-POF, *n* = 84) and the Non-AP group (*n* = 31), and between the POF (*n* = 35) and Non-POF (*n* = 49) subgroups, using Fisher’s exact test; *P* < 0.05 was considered statistically significant.

### 2.3 Microbiota analysis

Microbial community analyses were performed in R (v4.0.1) using mNGS-derived species relative abundance data. Alpha diversity was quantified with the Shannon, Simpson, ACE, and Chao1 indices. Pairwise differences in alpha-diversity indices were assessed using two-sided Wilcoxon rank-sum tests, with Bonferroni correction applied to the three pairwise comparisons within each index. Rank-biserial correlations and bootstrap 95% confidence intervals were reported as effect estimates. Beta diversity was assessed using Bray-Curtis dissimilarity, visualized by PCA and PCoA; overall between-group differences were tested using PERMANOVA (adonis function), with R^2^ reported as the effect size. Pairwise dissimilarity distributions were presented as box plots. Community composition was summarized at the genus and species levels using stacked bar charts and Venn diagrams of the top 20 taxa. Differential abundance across groups was tested by Kruskal-Wallis; for species with nominally significant overall differences, Dunn *post-hoc* tests were used for pairwise comparisons. *P*-values were adjusted using the Bonferroni method for the three pairwise comparisons within each species. Effect sizes were calculated as $r=Z/\sqrt{n_{1}+n_{2}}$, where *Z* is the Dunn test statistic and n_1_ and n_2_ are the sample sizes of the two compared groups. Spearman correlation was used to examine associations between microbial abundance and clinical variables. For species significantly associated with AP diagnosis, exploratory Firth penalized logistic regression was performed to assess the robustness of these associations after adjustment for key clinical factors. Clinical covariates included in the models were prespecified based on their clinical relevance, between-group comparisons of baseline characteristics, and their potential influence on microbial composition, microbial detection was coded as a binary variable (0 = absent, 1 = detected). Random forest classification models were constructed at six taxonomic levels (phylum, class, order, family, genus, and species) using the R package randomForest (ntree = 500, default mtry), with grouping label as the dependent variable and filtered microbial taxa as predictive features, evaluated by five repeats of 10-fold stratified cross-validation. Feature importance was quantified by mean decrease in accuracy and mean decrease in Gini impurity. The area under the receiver operating characteristic curve (AUC) with 95% confidence interval was calculated to evaluate discriminatory capacity.

### 2.4 Statistical analysis

Continuous variables are presented as median (interquartile range) and compared across groups using the Kruskal-Wallis test; categorical variables are presented as frequency (percentage) and compared using the chi-square test or Fisher’s exact test as appropriate. Where applicable, *P-*values from multiple pairwise comparisons were adjusted using the Bonferroni method. Firth penalized logistic regression results are reported as odds ratios (OR) with 95% confidence intervals (CI). Random forest model performance is reported as the area under the receiver operating characteristic curve (AUC) with 95% CI. All analyses were performed in R (v4.0.1); two-tailed *P* < 0.05 was considered statistically significant.

## 3 Results

### 3.1 Baseline clinical characteristics of the three groups

A total of 115 patients were enrolled: 35 in the POF group, 49 in the Non-POF group, and 31 in the Non-AP group. Baseline characteristics are summarized in Table 1. Among AP patients, the etiology of pancreatitis was comparable between the two groups, with biliary (POF 51.4%, Non-POF 55.1%) and hypertriglyceridemia-induced (POF 45.7%, Non-POF 42.9%) pancreatitis predominating in both. In the Non-AP group, intra-abdominal infections secondary to gastrointestinal perforation or gastrointestinal surgery were the most common (*n* = 23, 74.2%), with biliary tract-source secondary and spontaneous intra-abdominal infections accounting for the remainder (*n* = 4 each, 12.9% each).

**TABLE 1:** Baseline clinical characteristics of the three groups.

Variable	POF (*n* = 35)	Non-POF (*n* = 49)	Non-AP (*n* = 31)	*P*-value
Demographics				
Gender Male (n, %)	28 (80.0%)	33 (67.3%)	23 (74.2%)	0.43
Gender Female (n, %)	7 (20.0%)	16 (32.7%)	8 (25.8%)	0.43
Age (years, median, IQR)	51.0(34.0, 58.0)	42.0(35.0, 57.0)	69.0(60.0, 73.0)	<0.001
Etiology of pancreatitis, n (%)				
Hyperlipidemia	16(45.7%)	21 (42.9%)		
Biliary	18 (51.4%)	27(55.1%)		
Alcoholic	1 (2.9%)	0 (0%)		
Other	0 (0%)	1 (2.0%)		
Non-AP intra-abdominal infection etiology, n (%)				
GI perforation-associated			11 (35.5%)	
Post-operative gastrointestinal surgery-associated			12 (38.7%)	
Biliary tract-source secondary			4 (12.9%)	
Spontaneous intra-abdominal infection			4 (12.9%)	
Drainage modality, n(%)				0.524
Percutaneous drainage	11 (31.4%)	14 (28.6%)	10 (32.3%)	
Endoscopic transmural drainage	8 (22.9%)	5 (10.2%)	4 (12.9%)	
Surgical drainage	16 (45.7%)	30 (61.2%)	17 (54.8%)	
Sample collection, day, median (IQR)				
ICU admission-to-sampling interval	2.0 (1.0, 4.5)	2.0 (2.0, 4.0)	3.0 (2.0, 5.0)	0.279
Pre-sampling antibiotic duration	26.0 (19.0, 34.0)	32.0 (22.0, 36.0)	4.0 (2.0, 6.0)	< 0.001
Pre-sampling antibiotic classes, n(%)				
Empirical antibiotic use	35 (100%)	48 (98.0%)	31 (100%)	0.507
Beta-lactam	34 (97.1%)	48 (98.0%)	31 (100%)	1.000
Tetracycline	22 (62.9%)	24 (49.0%)	11 (35.5%)	0.092
Other	3 (8.6%)	8 (16.3%)	7 (22.6%)	0.298
Carbapenem	26 (74.3%)	28 (57.1%)	16 (51.6%)	0.135
Laboratory index, median (IQR)				
Leukocytes ( × 10^9^/L)	11.3(7.0,16.1)	11.1(7.2,15.3)	9.7(6.1,15.2)	0.522
CRP (mg/L)	148.0(98.0,216.0)	108.0(71.0,145.0)	137.0(78.0,192.0)	0.136
PCT (ng/mL)	3.0(1.1,15.2)	0.5(0.2,1.3)	3.5(1.3,22.4)	<0.001
Fibrinogen (g/L)	4.8(3.0,6.4)	4.5(3.2,5.6)	4.1(2.4,5.7)	0.212
Creatinine (μmol/L)	127.0(60.0,260.0)	49.0(38.5,75.0)	67.0(55.0,95.0)	<0.001
Clinical manifestations				
Temperature at sampling (°C)	38.6(38.2,39.2)	38.3(37.5,39.0)	38.3(37.8,38.9)	0.055
Gas bubble sign, n (%)	28 (80.0%)	44 (89.8%)	0 (0%)	<0.001
Complications, n (%)	22 (62.9%)	17 (34.7%)	5 (16.1%)	<0.001
Disease severity and comorbidities, median (IQR)				
CCI	3.0 (2.0, 6.0)	1.0 (1.0, 3.0)	4.0 (3.0, 6.0)	<0.001
APACHE II	21.0 (18.0, 25.0)	18.0 (16.0, 19.0)	19.0 (17.5, 23.5)	<0.001

*P-*values represent comparisons across all three groups. Etiology of pancreatitis was applicable only to AP patients (POF and Non-POF groups), and Non-AP intra-abdominal infection etiology was applicable only to the Non-AP group; no statistical comparisons were performed for these variables. The *P*-value for drainage modality represents the overall Fisher’s exact test result for the three-category distribution. Pre-sampling antibiotic classes are reported as independent utilization rates for each category; a single patient may be counted in more than one category, and *P*-values are based on independent Fisher’s exact tests for each category. POF, persistent organ failure; AP, acute pancreatitis; CRP, C-reactive protein; PCT, procalcitonin; CCI, Charlson Comorbidity Index; APACHE II, Acute Physiology and Chronic Health Evaluation II; IQR, interquartile range.

Sex distribution was balanced across groups (*P* = 0.43). The Non-AP group was significantly older than both pancreatitis groups (median 69.0 vs. 51.0 and 42.0 years; *P* < 0.001), consistent with the higher prevalence of gastrointestinal perforation in older adults. Pus collection modalities—CT/ultrasound-guided percutaneous drainage, endoscopic transmural drainage (EUS-TD), and surgical drainage—were comparably distributed across groups (*P* = 0.524). The ICU admission-to-sampling interval did not differ significantly among groups (*P* = 0.279), indicating comparable sampling time points. The overall empirical antibiotic utilization rate and pre-sampling antibiotic class distribution were comparable across groups (all *P* > 0.05; Table 1). Pre-sampling antibiotic duration, however, differed significantly (*P* < 0.001): both AP subgroups had substantially longer exposure than the Non-AP group (POF 26.0 days; Non-POF 32.0 days; Non-AP 4.0 days), reflecting the characteristically prolonged disease course of severe acute pancreatitis.

APACHE II scores differed significantly across groups (POF 21.0; Non-POF 18.0; Non-AP 19.0; *P* < 0.001), as did Charlson Comorbidity Index scores (POF 3.0; Non-POF 1.0; Non-AP 4.0; *P* < 0.001). Leukocyte count, CRP, and fibrinogen were comparable across groups (*P* = 0.522, 0.136, and 0.212, respectively), indicating a broadly similar systemic inflammatory state at sampling. PCT differed significantly among groups (*P* < 0.001), reflecting differences in infection severity and pathogen profiles across the three clinical entities. Creatinine was markedly elevated in the POF group (median 127.0 μmol/L; *P* < 0.001), consistent with active organ failure at the time of sampling. Temperature at sampling did not differ significantly across groups (*P* = 0.055). Gas bubble sign was present in the majority of AP patients (POF 80.0%, Non-POF 89.8%) and absent in all Non-AP patients (*P* < 0.001). Complication rates followed a stepwise pattern (POF 62.9%, Non-POF 34.7%, Non-AP 16.1%; *P* < 0.001).

### 3.2 Pathogen ecology and resistance gene burden in pancreatitis-associated versus non-pancreatitis intra-abdominal infection

#### 3.2.1 mNGS-based pathogen detection profiles

Overall mNGS pathogen positivity did not differ significantly across groups (POF 85.7%, Non-POF 93.9%, Non-AP 83.9%; combined AP 90.5%; AP vs. Non-AP *P* = 0.507; POF vs. Non-POF *P* = 0.268), whereas the composition of detected organisms differed substantially.

**FIGURE 2:**
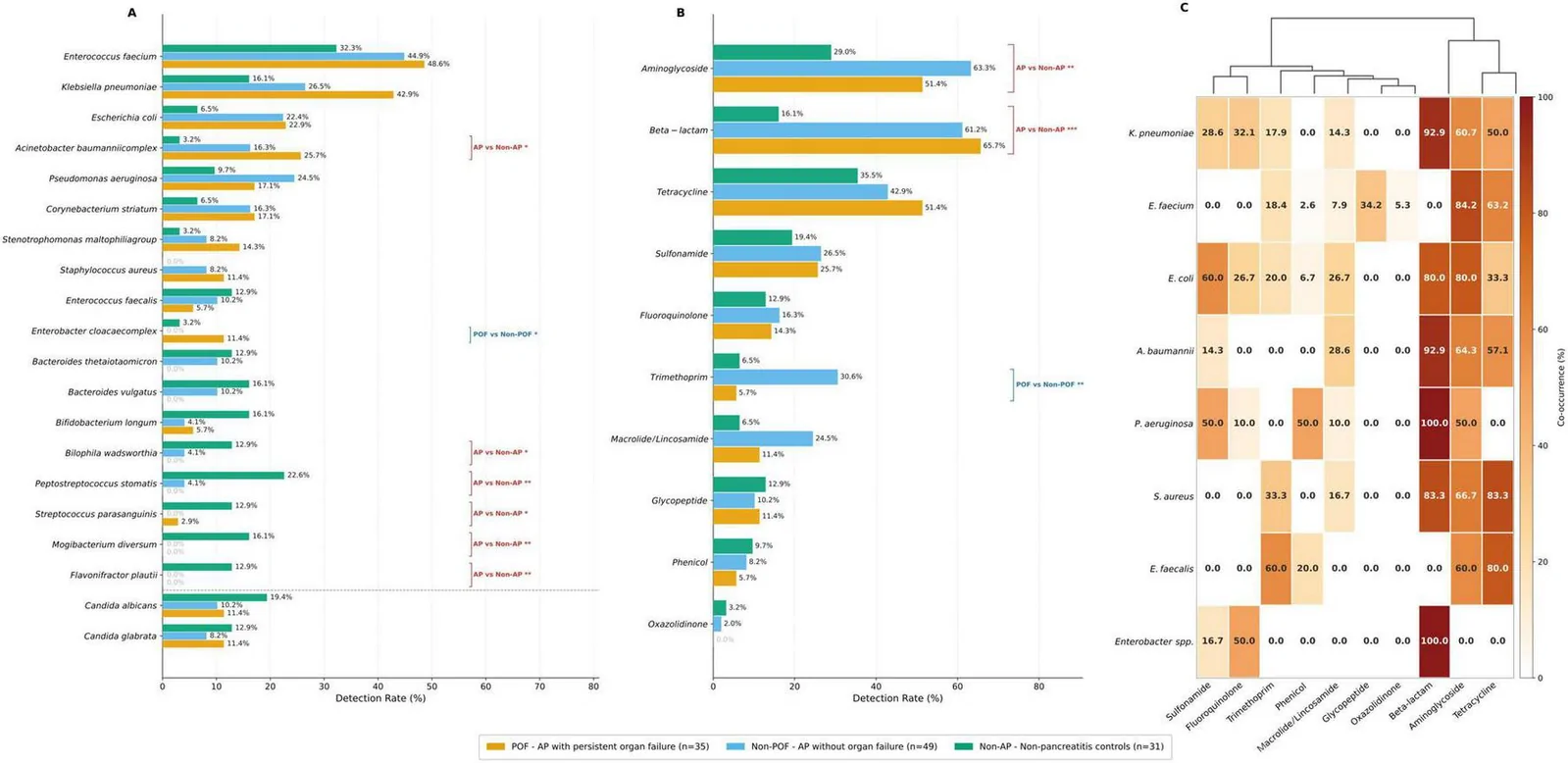
Pathogen detection spectrum and resistance gene profiles of pus samples across the three groups. **(A)** Detection rates of the top 20 microbial species across groups. **(B)** Detection rates of ARG categories across groups. **(C)** Heatmap of ARG co-detection rates (%) for eight predominant pathogens across ten ARG categories; color scale ranges from white to dark red representing co-detection rates from 0 to 100%; numerical values indicate co-detection rates (%). Statistical comparisons were performed at two levels: between the combined AP group (POF + Non-POF) and the Non-AP group, and between the POF and Non-POF subgroups; asterisks indicate significant differences. *P < 0.05, **P < 0.01, ***P < 0.001. POF, persistent organ failure; AP, acute pancreatitis; ARG, antimicrobial resistance gene.

Both pancreatitis groups shared a convergent pathogen profile dominated by ICU-associated organisms (Figure 2A): *Enterococcus faecium* (POF 48.6%, Non-POF 44.9%), *K. pneumoniae* (POF 42.9%, Non-POF 26.5%), *Acinetobacter baumannii* complex (POF 25.7%, Non-POF 16.3%), and *P. aeruginosa* (POF 17.1%, Non-POF 24.5%) were among the most frequently detected species in both groups. *A. baumannii* complex was detected at a higher rate in the combined AP group than in Non-AP patients (*P* < 0.05). Differences between the two pancreatitis subgroups were limited; *Enterobacter cloacae* complex was the only species showing a significant difference (POF 11.4% vs. Non-POF 0%; *P* < 0.05), and no other dominant pathogen differed significantly between them.

The Non-AP group harbored a distinct pathogen profile, with substantially higher detection rates of intestinal mucosal-associated and oropharyngeal commensal organisms: *Peptostreptococcus stomatis* (22.6% vs. < 5% in AP; *P* < 0.01), *Mogibacterium diversum* (16.1% vs. 0%; *P* < 0.01), *Bilophila wadsworthia* (12.9% vs. < 5%; *P* < 0.05), *Streptococcus parasanguinis* (12.9% vs. < 3%; *P* < 0.05), and *Flavonifractor plautii* (12.9% vs. 0%; *P* < 0.01). *Candida* spp. were detected in approximately 10% of patients across all groups with no significant differences. In summary, the compositional contrast was most pronounced between pancreatitis and non-pancreatitis patients—ICU-associated pathogens dominating in the former and commensal organisms prevailing in the latter—while differences between the two pancreatitis subgroups were largely confined to individual species.

#### 3.2.2 mNGS-based ARG detection profiles

ARG positivity was markedly higher in AP patients than in Non-AP controls (69.0% vs. 45.2%; *P* = 0.029), whereas no difference was observed between the POF and Non-POF groups (68.6% vs. 69.4%; *P* = 1.000), indicating that ARG positivity was associated with AP status but not with POF status in this cross-sectional cohort.

At the ARG category level (Figure 2B), Beta-lactam (POF 65.7%, Non-POF 61.2% vs. Non-AP 16.1%; *P* < 0.001) and Aminoglycoside (POF 51.4%, Non-POF 63.3% vs. Non-AP 29.0%; *P* < 0.01) resistance genes were significantly enriched in AP patients relative to Non-AP controls. Subgroup analysis of Beta-lactam ARG (Supplementary Figure 1) showed significant AP-versus-Non-AP differences across all subcategories: Carbapenemase (POF 34.3%, Non-POF 30.6% vs. Non-AP 6.5%; *P* < 0.01), ESBL (POF 42.9%, Non-POF 22.4% vs. Non-AP 9.7%; *P* < 0.05), AmpC (POF 22.9%, Non-POF 26.5% vs. Non-AP 6.5%; *P* < 0.05), and Penicillinase/Others (POF 40.0%, Non-POF 32.7% vs. Non-AP 12.9%; *P* < 0.05), with the Carbapenemase subgroup showing the most pronounced difference. Sulfonamide, Fluoroquinolone, and Glycopeptide resistance gene rates did not differ significantly across the three groups. Within the AP population, ARG category rates were comparable between POF and Non-POF subgroups across nearly all categories, including all Beta-lactam subcategories. The sole exception was Trimethoprim resistance genes, which were more frequent in Non-POF than POF patients (30.6% vs. 5.7%; *P* < 0.01).

#### 3.2.3 Pathogen–ARG co-occurrence analysis

Resistance-gene detection profiles varied substantially across pathogen species (Figure 2C), with *K. pneumoniae* and *E. faecium* showing the broadest resistance-gene profiles.

Among Gram-negative pathogens, Beta-lactam resistance genes were frequently detected in *K. pneumoniae*-positive samples (92.9%), with carbapenemase-related genes detected in 18 of 28 samples (64.3%). Culture-based carbapenem susceptibility data were available for 18 culture-positive *K. pneumoniae* isolates, including 14 CRKP isolates (77.8%). In an exploratory genotype–phenotype comparison, 14 cases met the mNGS–culture pairing criteria. Carbapenemase-related genes were detected by mNGS in 9 of 11 culture-confirmed CRKP cases (81.8%) but in none of the three carbapenem-susceptible cases (Supplementary Table 1), indicating partial concordance between mNGS ARG detection and phenotypic carbapenem resistance in *K. pneumoniae*. Other Gram-negative pathogens, including *E. coli*, *A. baumannii*, *P. aeruginosa*, and *Enterobacter* spp., also showed frequent detection of Beta-lactam resistance genes.

Among Gram-positive pathogens, *E. faecium*-positive samples frequently harbored aminoglycoside- (84.2%), tetracycline- (63.2%), and glycopeptide-resistance genes (34.2%). *S. aureus* and *E. faecalis* showed distinct resistance-gene profiles, with frequent detection of Beta-lactam/tetracycline resistance genes in *S. aureus* and tetracycline/aminoglycoside resistance genes in *E. faecalis*.

Overall, the resistance-gene landscape was characterized by broad ARG detection among ICU-associated pathogens, particularly *K. pneumoniae* and *E. faecium*. Integrated interpretation of mNGS-based ARG profiling with available phenotypic susceptibility data may provide complementary information for resistance-risk assessment and empirical antimicrobial decision-making.

### 3.3 Microbial community characterization of pus samples across the three groups

Having characterized pathogen detection profiles and ARG burden, we next examined overall microbial community structure and diversity to delineate the ecological features of pancreatitis-associated intra-abdominal infection at the community level.

#### 3.3.1 Overall microbial community structure and diversity

**FIGURE 3:**
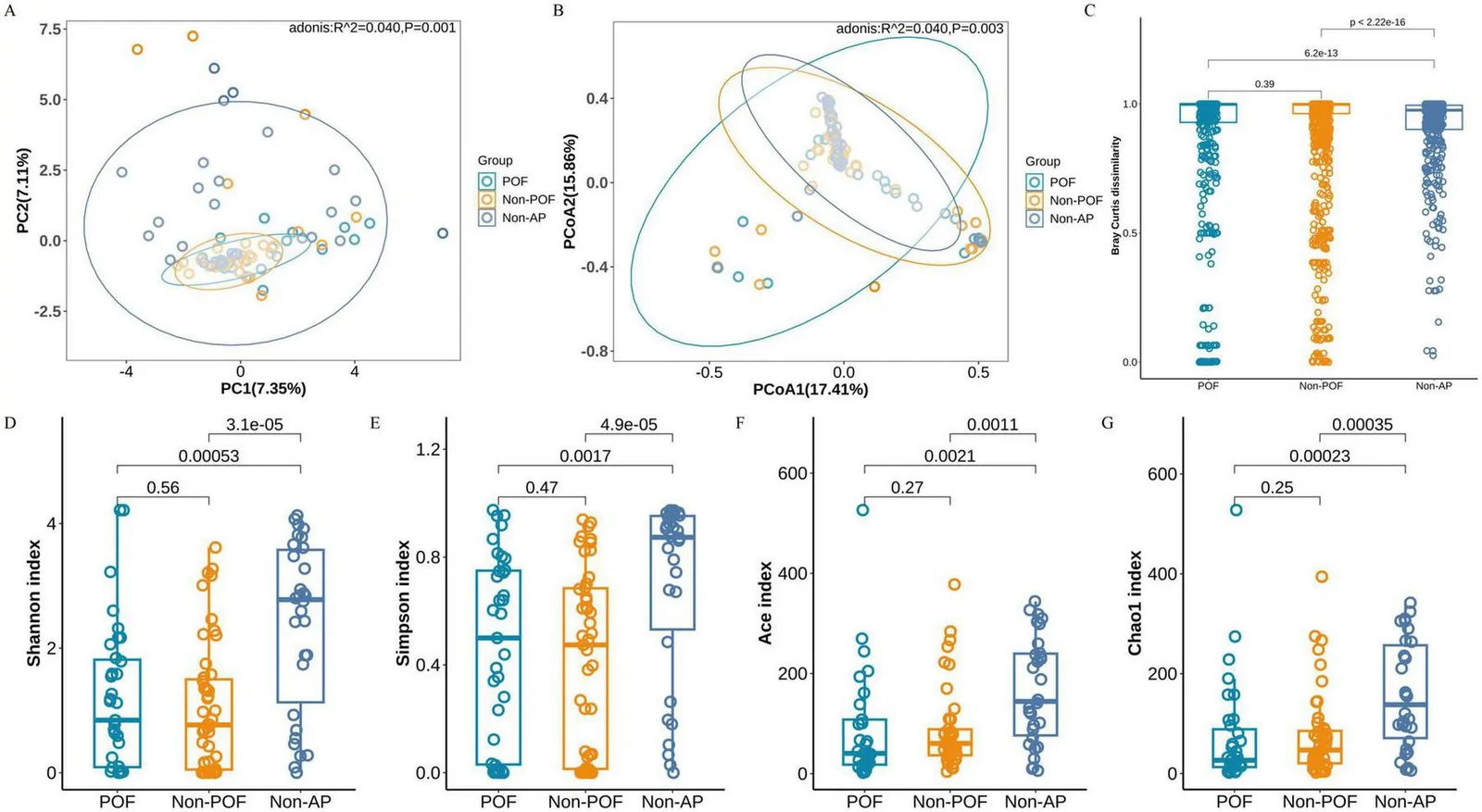
Microbial community structure and alpha diversity of pus samples across the three groups. **(A)** PCA scatter plot of microbial community composition. **(B)** PCoA scatter plot based on Bray-Curtis dissimilarity. **(C)** Pairwise Bray-Curtis dissimilarity among the three groups. **(D–G)** Comparisons of the Shannon index **(D)**, Simpson index **(E)**, ACE index **(F)**, and Chao1 index **(G)** across the three groups. *P*-values indicate pairwise comparisons.

Both PCA (Figure 3A; *P* = 0.001, *R*^2^ = 0.04) and PCoA (Figure 3B; *P* = 0.003, *R*^2^ = 0.04) showed clear compositional separation among the three groups, confirmed by PERMANOVA. Pairwise comparisons revealed marked structural differences between both pancreatitis groups and the Non-AP group (POF vs. Non-AP: *P* = 6.2 × 10^−13^; Non-POF vs. Non-AP: *P* = 2.22 × 10^−16^), whereas community composition did not differ between POF and Non-POF (*P* = 0.39; Figure 3C). To account for the potential confounding effect of organ dysfunction present in 41.9% of Non-AP patients, stratified pairwise comparisons were performed; significant differences persisted in both comparisons by Bray curtis dissimilarity (POF vs. Non-AP-OF/Non-AP-NOF: *P* = 0.0077/1.8 × 10^−11^; Non-POF vs. Non-AP-OF/Non-AP-NOF: *P* = 0.00077/2.22 × 10^−16^; Supplementary Figure 2), suggesting that the observed community divergence may reflect the pancreatitis disease process rather than organ failure status.

All four alpha diversity indices revealed overall differences across groups. Both pancreatitis groups had substantially reduced species richness (ACE: POF vs. Non-AP *P* = 0.0021, Non-POF vs. Non-AP *P* = 0.0011; Chao1: *P* = 0.00023 and *P* = 0.00035) and lower community diversity (Shannon: *P* = 0.00053 and *P* = 3.1 × 10^−5^; Simpson: *P* = 0.0017 and *P* = 4.9 × 10^−5^) relative to Non-AP patients (Figures 3D–G). None of the four indices differed between POF and Non-POF (all *P* > 0.05). After Bonferroni correction for the three pairwise comparisons within each alpha-diversity index, all differences between the two AP groups and the Non-AP group remained statistically significant, whereas no comparison between the POF and Non-POF groups reached statistical significance. Adjusted *P*-values, effect sizes, and their 95% confidence intervals are provided in Supplementary Table 2. These results indicate that community diversity was considerably reduced in both pancreatitis groups compared with Non-AP patients, a pattern that was not further stratified by organ failure status at sampling.

In a sensitivity analysis restricted to Non-AP patients with gastrointestinal perforation-associated or postoperative gastrointestinal surgery-associated infection (*n* = 23), the primary diversity patterns were preserved: both AP groups remained distinct from the restricted Non-AP subgroup in beta-diversity analyses and exhibited lower alpha diversity, whereas no significant alpha- or beta-diversity differences were observed between the POF and Non-POF groups (Supplementary Figure 3).

#### 3.3.2 Species composition and differential species analysis

**FIGURE 4:**
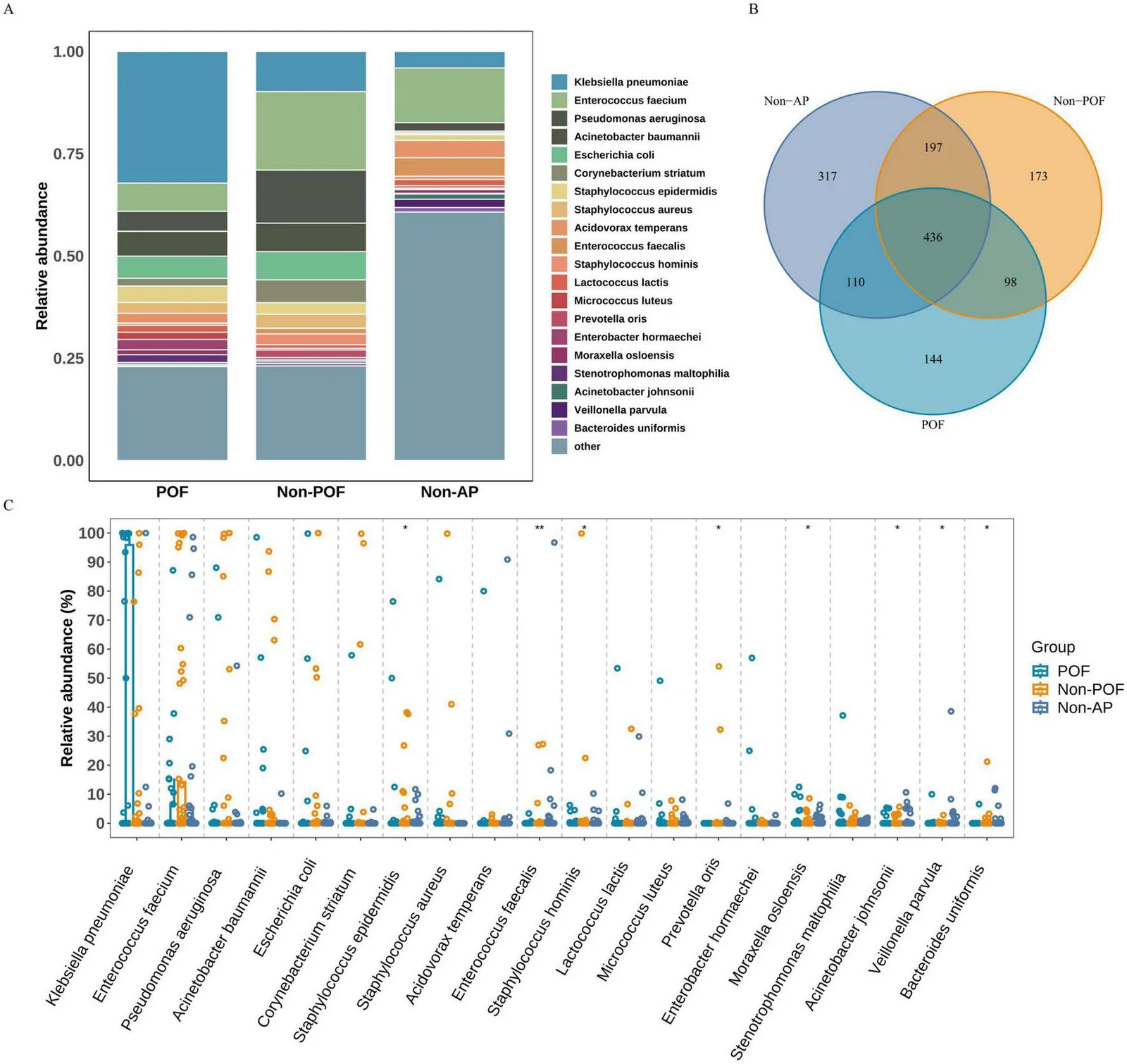
Species-level community composition and differential species analysis of pus samples across the three groups. **(A)** Stacked bar chart of top 20 species by mean relative abundance across groups; “other” denotes the cumulative abundance of taxa beyond the top 20. **(B)** Venn diagram of shared and group-exclusive species across the three groups. **(C)** Between-group comparisons of relative abundance for the top 20 species. *P < 0.05, **P < 0.01.

At the species level (Figure 4A), *K. pneumoniae* was the dominant species in the POF group (32.16%), whereas the Non-POF group showed a more balanced co-dominance of *K. pneumoniae*, *P. aeruginosa*, and *E. faecium*; Non-AP pus was predominantly composed of “other” taxa (60.76%). Venn diagram analysis (Figure 4B) identified 98 species shared exclusively between the two pancreatitis groups and absent from Non-AP samples, representing candidate taxa for an AP-associated microbial pattern. Genus-level analysis yielded consistent findings (Supplementary Figure 4): both POF and Non-POF groups showed marked enrichment of *Klebsiella*, *Enterococcus*, *Pseudomonas*, and *Acinetobacter*, while the Non-AP group had considerably lower abundances of these genera with dispersed low-abundance taxa predominating.

Kruskal-Wallis testing across the top 20 species identified eight differentially abundant species (Figure 4C): *Staphylococcus epidermidis*, *E. faecalis*, *Staphylococcus hominis*, *Prevotella oris*, *Moraxella osloensis*, *Acinetobacter johnsonii*, *Veillonella parvula*, and *Bacteroides uniformis*. All eight were more abundant in Non-AP than in either pancreatitis group, a direction opposite to the enrichment pattern of high-risk pathogens. Bonferroni-adjusted Dunn post-hoc comparisons showed that *E. faecalis* was more abundant in the Non-AP group than in both the POF and Non-POF groups (both *P*_*adj*_ < 0.01). *S. epidermidis*, *S. hominis*, and *M. osloensis* were more abundant in the Non-AP group than in the Non-POF group only, whereas *P. oris*, *A. johnsonii*, *V. parvula*, and *B. uniformis* were more abundant in the Non-AP group than in the POF group only (all *P*_*adj*_ < 0.05). The remaining pairwise comparisons did not retain statistical significance after correction, although their abundance patterns were directionally consistent with relative enrichment in the Non-AP group. No pairwise comparison between the POF and Non-POF groups remained statistically significant after Bonferroni correction (Table 2). These species-level findings should be interpreted as exploratory and are consistent with the broader community-level differences observed between AP-associated and non-pancreatitis pus.

**TABLE 2:** Exploratory Bonferroni-adjusted pairwise comparisons of selected species among the three patient groups.

Species	Non-AP vs. Non-POF *P*(r)	Non-AP vs. POF *P*(r)	Non-POF vs. POF *P*(r)
*Staphylococcus epidermidis*	0.0411* (0.276)	0.0679 (0.281)	1.0000 (–0.004)
*Enterococcus faecalis*	0.0094** (0.330)	0.0058** (0.382)	1.0000 (0.040)
*Staphylococcus hominis*	0.0173* (0.309)	0.0937 (0.265)	1.0000 (–0.053)
*Prevotella oris*	0.8544 (0.120)	0.0438* (0.301)	0.3274 (0.175)
*Moraxella osloensis*	0.0196* (0.304)	0.0989 (0.262)	1.0000 (–0.051)
*Acinetobacter johnsonii*	0.0896 (0.243)	0.0270* (0.322)	1.0000 (0.070)
*Veillonella parvula*	0.0770 (0.249)	0.0419* (0.303)	1.0000 (0.044)
*Bacteroides uniformis*	0.6805 (0.135)	0.0248* (0.325)	0.2798 (0.183)

Pairwise comparisons were performed using Dunn’s test following Kruskal-Wallis testing. Values are presented as Bonferroni-adjusted two-sided *P*-values (*P*_*adj*_) and effect sizes r, calculated as $r=Z/\sqrt{n_{1}+n_{2}}$. Positive r values indicate higher mean ranks in the first-listed group. *P*_*adj*_ < 0.05 and *P*_*adj*_ < 0.01 are indicated by * and **, respectively. POF, persistent organ failure; AP, acute pancreatitis.

#### 3.3.3 Correlation analysis between pus microbiota and clinical parameters

**FIGURE 5:**
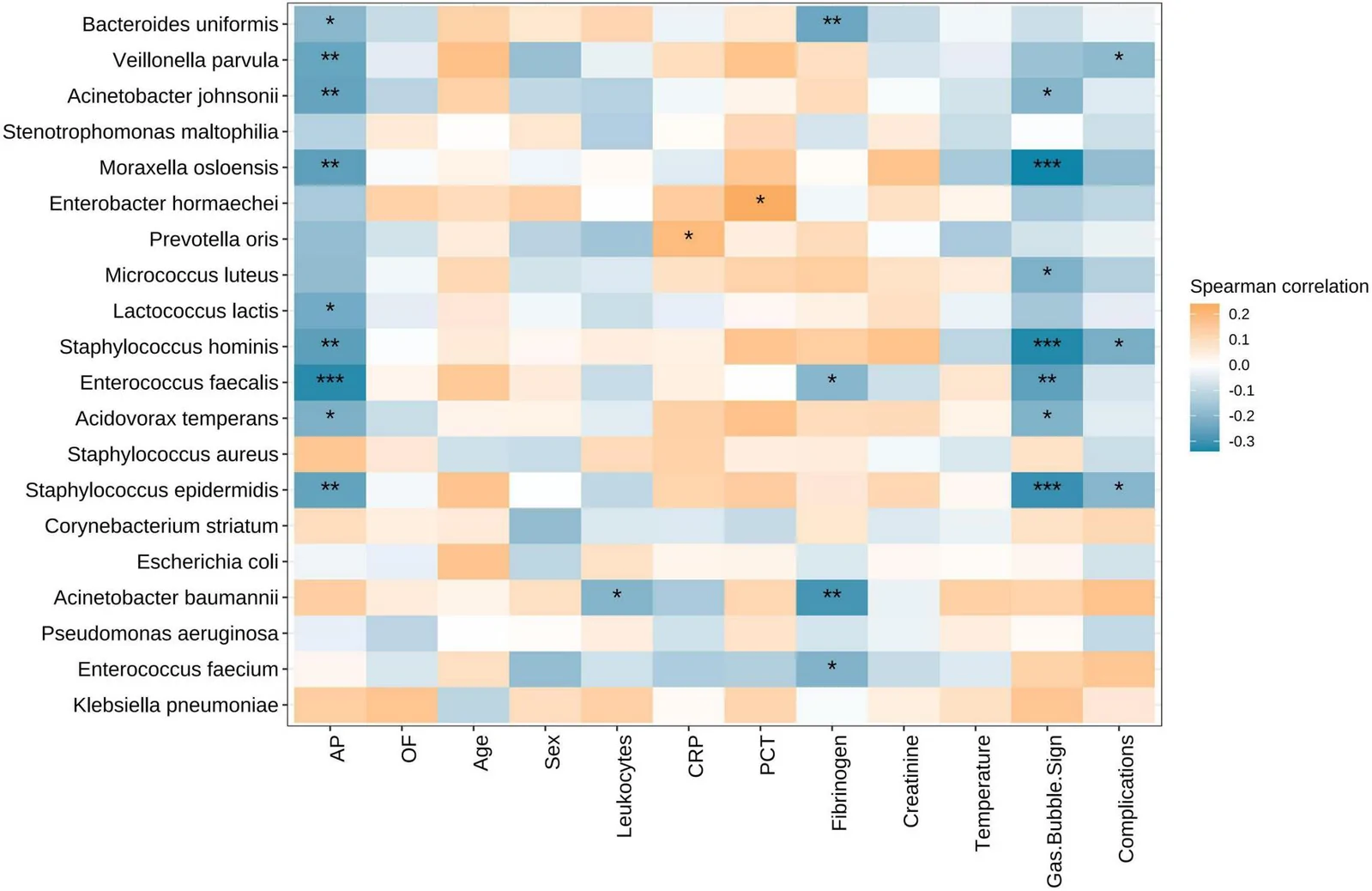
Spearman correlation heatmap between the top 20 pus microbial species and clinical parameters including disease classification. Colors represent Spearman correlation coefficients; orange indicates positive correlation and blue indicates negative correlation, with color intensity reflecting the magnitude of correlation. *P < 0.05, **P < 0.01, ***P < 0.001.

Spearman correlation analysis was conducted between the top 20 species and 12 clinical variables (Figure 5). Several microbial taxa showed associations with inflammatory markers, clinical features, and AP status. Notably, eight species, including *E. faecalis*, *V. parvula*, *S. hominis*, A. *johnsonii*, *S. epidermidis* (all *P* < 0.01) and *B. uniformis*, *Lactococcus lactis*, A. temperans (all *P* < 0.05), showed inverse associations with AP diagnosis, whereas no species showed a meaningful correlation with organ failure status, consistent with the lack of community-level differences between POF and Non-POF groups.

To assess the robustness of species-level associations with AP status after adjustment for key clinical factors, exploratory Firth penalized logistic regression analyses were performed for the eight species inversely associated with AP status in the correlation analysis. Covariates were selected based on between-group baseline differences and clinical relevance, including CCI, APACHE II score, pre-sampling antibiotic duration, and ICU admission-to-sampling interval. Models were adjusted separately for each clinical factor, with the corresponding microbial detection variable and age included in each model. *E. faecalis* consistently remained inversely associated with AP status across all adjusted models (all *P* ≤ 0.006; Table 3). *S. hominis*, *M. osloensis*, and *A. johnsonii* retained inverse associations after adjustment for CCI, APACHE II score, or ICU admission-to-sampling interval, but these associations were attenuated after adjustment for pre-sampling antibiotic duration. The remaining species showed no significant associations after adjustment. Given the limited sample size and potential residual confounding, these findings should be interpreted as exploratory and require confirmation in larger prospective cohorts.

**TABLE 3:** Exploratory Firth penalized logistic regression analyses of selected microbial species in relation to AP status.

Species	Adjustment model	OR	95%CI	*P*-value
*Enterococcus faecalis*	Age + CCI	0.204	0.062–0.614	0.004**
	Age + APACHE II	0.216	0.066–0.647	0.006**
	Age + pre-sampling antibiotic duration	0.079	0.008–0.440	0.003**
	Age + ICU admission-to-sampling interval	0.216	0.066–0.649	0.006**
*Staphylococcus hominis*	Age + CCI	0.255	0.086–0.708	0.009**
	Age + APACHE II	0.251	0.084–0.697	0.008**
	Age + pre-sampling antibiotic duration	0.657	0.167–2.624	0.545
	Age + ICU admission-to-sampling interval	0.221	0.071–0.635	0.005**
*Moraxella osloensis*	Age + CCI	0.328	0.114–0.886	0.028*
	Age + APACHE II	0.334	0.116–0.900	0.030*
	Age + pre-sampling antibiotic duration	0.535	0.132–2.075	0.363
	Age + ICU admission-to-sampling interval	0.331	0.116–0.891	0.028*
*Acinetobacter johnsonii*	Age + CCI	0.347	0.120–0.946	0.039*
	Age + APACHE II	0.359	0.125–0.972	0.044*
	Age + pre-sampling antibiotic duration	0.582	0.148–2.246	0.428
	Age + ICU admission-to-sampling interval	0.369	0.130–0.996	0.049*

AP status was the dependent variable (0 = Non-AP, 1 = AP). Each model included the corresponding binary microbial detection variable, age, and one additional prespecified clinical covariate entered separately: CCI, APACHE II score, pre-sampling antibiotic duration, or ICU admission-to-sampling interval. Firth penalized logistic regression was used because of the limited complete-case sample size and sparse detection of some species. ORs represent the association between detection of the corresponding species and AP status. Only species with a statistically significant association in at least one adjusted model are presented; the remaining selected species showed no statistically significant associations with AP status in any adjusted model. CCI, Charlson Comorbidity Index; APACHE II, Acute Physiology and Chronic Health Evaluation II; AP, acute pancreatitis; ICU, intensive care unit; OR, odds ratio; CI, confidence interval. *P < 0.05, **P < 0.01.

#### 3.3.4 Exploratory random forest predictive models based on multi-level taxonomic features of pus microbiota

**FIGURE 6:**
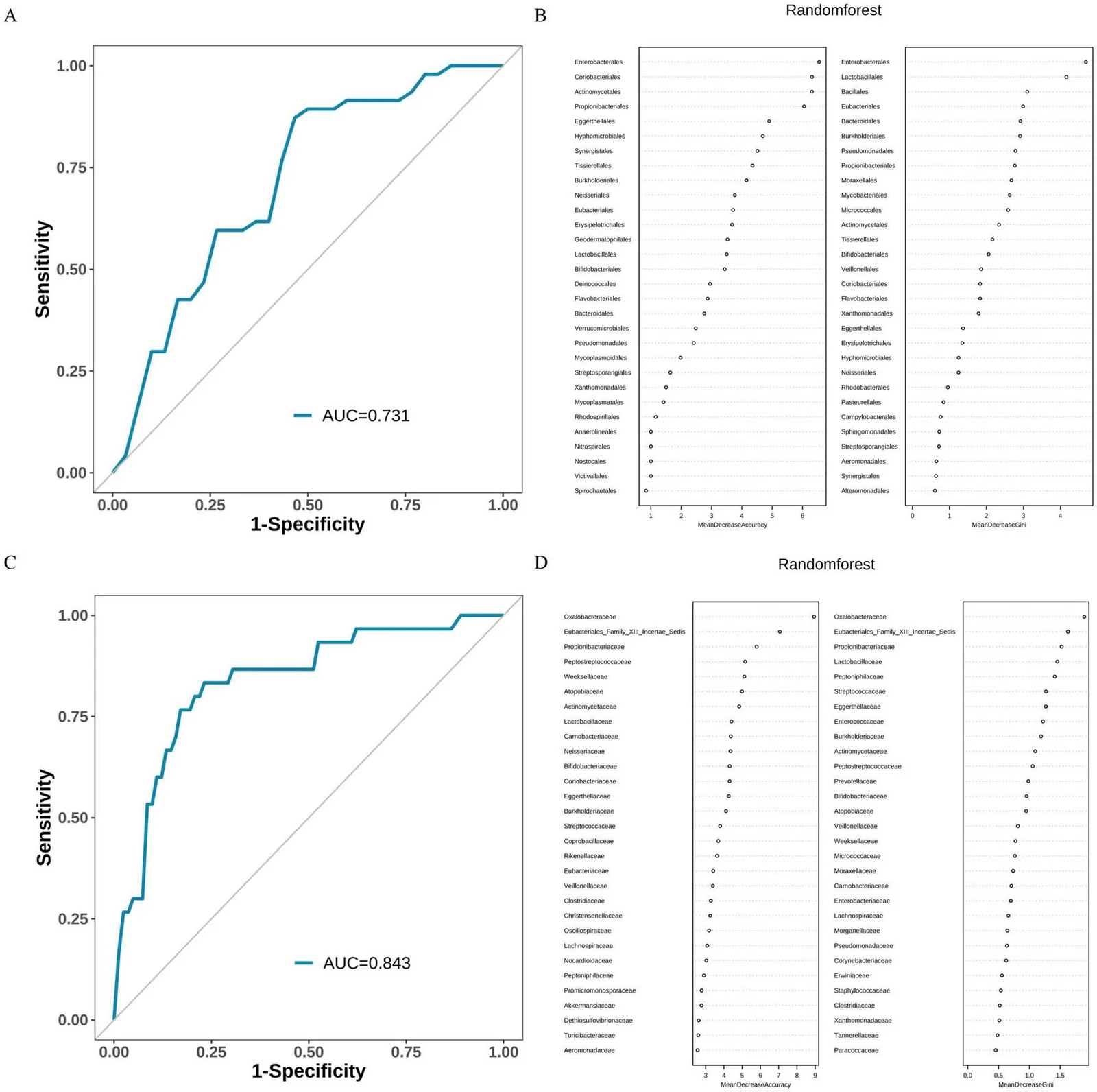
Random forest discriminatory models based on multi-level taxonomic features of pus microbiota. **(A)** ROC curve of the three-group classification model at the order level. **(B)** Feature importance plots for the three-group model at the order level. **(C)** ROC curve of the pancreatitis identification model at the family level. **(D)** Feature importance plots for the pancreatitis identification model at the family level. All AUC values reflect within-cohort discriminatory capacity evaluated by internal cross-validation and should be interpreted as exploratory findings pending external independent validation.

Random forest models were constructed at six taxonomic levels to evaluate the exploratory capacity of pus microbial features to discriminate between patient groups. All AUC values reported below reflect within-cohort discriminatory performance derived from internal cross-validation and should be interpreted as exploratory findings, as no external independent validation cohort was available in this study. For the three-group classification task (POF vs. Non-POF vs. Non-AP), AUC values ranged from 0.587 to 0.731 across taxonomic levels, with the order-level model performing best (AUC = 0.731); *Enterobacterales* ranked first in feature importance by both mean decrease in accuracy and mean decrease in Gini impurity (Figures 6A,B and Table 4).

**TABLE 4:** Performance summary of exploratory random forest predictive models based on multi-level taxonomic features of pus microbiota.

Model	Level	AUC	95%CI	Threshold	Sensitivity	Specificity
Three-group	Phylum	0.587	0.456–0.717	0.446	46.80%	83.30%
Three-group	Class	0.687	0.565–0.810	0.48	53.20%	80.00%
Three-group	Order	0.731	0.612–0.850	0.298	87.20%	56.70%
Three-group	Family	0.705	0.582–0.828	0.464	63.80%	76.70%
Three-group	Genus	0.691	0.565–0.817	0.338	85.10%	53.30%
Three-group	Species	0.7	0.579–0.821	0.387	76.60%	63.30%
AP identification	Phylum	0.769	0.665–0.873	0.282	73.30%	74.40%
AP identification	Class	0.841	0.753–0.930	0.271	80.00%	80.50%
AP identification	Order	0.831	0.736–0.926	0.259	83.30%	76.80%
AP identification	Family	0.843	0.758–0.929	0.255	83.30%	78.00%
AP identification	Genus	0.811	0.716–0.906	0.19	83.30%	73.20%
AP identification	Species	0.8	0.708–0.892	0.366	63.30%	87.80%

Two classification tasks were evaluated: three-group classification (POF vs. Non-POF vs. Non-AP) and pancreatitis identification (AP vs. Non-AP). Models were constructed and evaluated at six taxonomic levels. AUC values are presented with 95% confidence intervals. The threshold represents the optimal classification cutoff determined by the Youden index. POF, persistent organ failure; AP, acute pancreatitis; AUC, area under the receiver operating characteristic curve; CI, confidence interval.

Given the structural similarity between POF and Non-POF communities demonstrated across preceding analyses, models targeting pancreatitis identification (AP vs. Non-AP) were constructed at the same six levels. AUC values exceeded those of the three-group models at every taxonomic level, ranging from 0.769 to 0.843. The family-level model achieved the highest discriminatory performance (AUC = 0.843, 95% CI: 0.758–0.929), with *Oxalobacteraceae* and *Propionibacteriaceae* ranking as the top features by both importance metrics (Figures 6C,D and Table 4). The consistently higher AUC values of the pancreatitis identification models across all taxonomic levels corroborate the community-level findings and indicate that the microbial composition of AP-associated pus carries quantifiable discriminatory information relative to non-pancreatitis abdominal infection.

## 4 Discussion

Metagenomic sequencing of 115 intra-abdominal pus samples from a surgical ICU yielded three principal findings. The pathogen composition of AP-associated pus differed substantially from that of non-pancreatitis abdominal infection, with ICU-adapted organisms dominating the former and commensal flora prevailing in the latter. Resistance gene burden was higher in AP patients, with carbapenemase genes detected at a considerably greater frequency than in non-pancreatitis controls. The pancreatitis-associated microbial profile was already established in patients with intact organ function at sampling and showed no meaningful difference between patients with and without POF at sampling, a pattern more consistent with microbial patterns associated with acute pancreatitis than with organ dysfunction as the primary driver. These findings provide an initial microbiome-level characterization of AP-associated intra-abdominal infection that may provide microbiological context relevant to empirical antimicrobial considerations in critically ill patients with suspected IPN.

Differences in the route and clinical context of infection may contribute to the compositional divergence between AP-associated and non-pancreatitis pus. The Non-AP group was clinically defined to include non-pancreatic intra-abdominal infections of heterogeneous origins, although gastrointestinal perforation-associated and postoperative gastrointestinal surgery-associated infections accounted for most cases. In infections involving gastrointestinal disruption, direct entry of luminal flora into the peritoneal cavity may contribute to the predominance of oral- and gut-derived commensals, including *Peptostreptococcus stomatis*, *Mogibacterium diversum*, and *Flavonifractor plautii*, observed in Non-AP pus. This pattern is consistent with microbial profiles reported in perforation-associated peritonitis (Wang and Yao, 2025; Blot et al., 2019), but should be interpreted in the context of the heterogeneous Non-AP comparator. In IPN, pathogens are thought to reach peripancreatic necrotic tissue primarily through enteric bacterial translocation across a disrupted intestinal barrier (Cui et al., 2025; Zhang et al., 2025). This process occurs in the context of prolonged ICU care, repeated broad-spectrum antibiotic exposure, and systemic immune impairment, which may favor the predominance of ICU-associated organisms with frequent resistance-gene detection and contribute to the convergent pathogen profile observed across both AP groups, characterized by *K. pneumoniae*, *E. faecium*, *E. coli*, *A. baumannii* complex, and *P. aeruginosa*. Hong et al. reported frequent detection of *A. baumannii*, *K. pneumoniae*, *E. faecalis*, and *E. coli* by blood mNGS in patients with IPN; subsequent mNGS-culture cross-validation of fine-needle aspiration specimens further identified *K. pneumoniae* and *E. faecium* as predominant detected pathogens (Hong et al., 2022; Hong et al., 2024). These previous findings are broadly consistent with the pathogen profile observed in the present study. Our findings extend these observations at the community level: compared with Non-AP group, AP-associated pus was characterized by dominance of ICU-associated pathogens, reduced alpha diversity, and relative depletion of commensal taxa. The 98 species shared exclusively by the two AP groups and absent from Non-AP samples further illustrate this community-level separation. The more frequent detection of *A. baumannii* complex in AP patients than in Non-AP controls from the same ICU is compatible with its recognized capacity to expand under prolonged hospitalization and antibiotic pressure (Makwana et al., 2026). However, these cross-sectional data cannot distinguish the relative contributions of infection source, antimicrobial exposure, ICU-related factors, and host condition to the observed microbial patterns.

The comparable fungal detection rates across all three groups (approximately 10%) aligned with the 11% rate reported by Wang et al. using mNGS in 420 peritonitis patients (Wang et al., 2025), suggesting that *Candida* spp. were not enriched in AP-associated pus in this cohort and that antifungal coverage decisions should be guided by host immune status and established risk factors rather than the diagnosis of pancreatitis alone.

Resistance gene data offered additional molecular-level insight into the antimicrobial challenge posed by AP-associated abdominal infection. ARG positivity was higher in AP patients than in non-pancreatitis controls, with Beta-lactam and Aminoglycoside resistance genes showing the most pronounced enrichment. Differences between the AP and Non-AP groups spanned all Beta-lactam subcategories—Carbapenemase, ESBL, AmpC, and Penicillinase/Others—with the Carbapenemase subgroup showing the greatest disparity. Carbapenemase resistance genes were co-detected in 64.3% (18/28) of *K. pneumoniae*-positive samples. Because the yield of conventional culture was limited, phenotypic susceptibility data were available for only 18 culture-positive *K. pneumoniae* isolates in this cohort, of which 14 (77.8%) were CRKP. Given the small number of isolates with available phenotypic susceptibility data, neither result can be interpreted as the prevalence of CRKP in this cohort. Validation in a larger cohort is therefore required. Nevertheless, national surveillance data provide relevant context. Liu et al. reported that CRKP accounted for 6.4–16.7% of clinical *K. pneumoniae* isolates between 2020 and 2024, while concurrent CHINET figures ranged from 20.8 to 24.8% (Liu L. et al., 2025). In comparison, the present cohort showed a high burden of carbapenemase-related gene detection in *K. pneumoniae*-associated samples and a high proportion of CRKP among culture-positive *K. pneumoniae* isolates. A plausible explanation lies in the clinical trajectory of severe AP itself: prolonged hospitalization, repeated broad-spectrum antibiotic courses, and frequent invasive procedures may collectively create selective conditions favoring the acquisition and local dissemination of resistant organisms, consistent with ICU admission, immunosuppression, and mechanical ventilation being established risk factors for CRKP infection (Özçelik et al., 2026; Jin et al., 2026). Non-AP patients with gastrointestinal perforation generally underwent earlier surgical source control and had shorter disease courses, which may have limited their cumulative exposure to resistance-selecting pressures and partly accounts for the lower carbapenemase gene detection rates in that group. Glycopeptide resistance genes were co-detected in 34.2% of *E. faecium*-positive samples, suggesting a notable molecular burden of glycopeptide-resistance determinants. Because of the low culture yield and the retrospective study design, paired phenotypic susceptibility data for *Enterococcus* isolates were largely unavailable; these findings therefore reflect gene-level detection rather than phenotypically confirmed VRE. Nevertheless, this proportion is broadly consistent with rising VRE rates documented in ICUs domestically and internationally (Liu L. et al., 2025; Biswas et al., 2026; Saharman et al., 2021). Taken together, these findings suggest that critically ill AP patients with suspected IPN may have an increased risk of infection involving resistant organisms. Empirical antimicrobial selection should therefore be guided by local epidemiological data, prior antimicrobial exposure, illness severity, source control, and culture-based susceptibility testing when available. In patients with negative cultures or while susceptibility results are pending, mNGS-derived ARG findings may provide complementary information for early resistance-risk assessment. The lower carbapenemase-gene detection burden observed in non-pancreatitis intra-abdominal infection from the same ICU further supports consideration of infection source and patient-specific risk factors when selecting empirical therapy. These observations require confirmation in larger prospective studies.

A notable observation in this study is that the pancreatitis-associated microbial profile in peripancreatic pus was already detectable in patients with intact organ function at sampling, and within this cross-sectional cohort, the presence or absence of POF showed no significant association with further differences in community structure. Five analytical approaches—beta diversity, alpha diversity, differential species abundance, logistic regression, and organ-function-matched pairwise comparisons—showed broadly consistent patterns. Stratified comparisons accounting for organ-function status in the Non-AP group further indicated that the observed microbial divergence remained associated with AP status; however, these cross-sectional comparisons cannot establish whether pancreatitis itself caused the observed differences. It bears emphasis that the POF and Non-POF groups represent cross-sectional snapshots of patients at different organ function states at sampling, not longitudinal observations within the same individuals; accordingly, these data do not permit the inference that organ failure progression leaves community dynamics unchanged over time, and this question will require prospective longitudinal sampling to resolve.

At the mechanistic level, two upstream pathological events that may antedate organ failure—intestinal barrier disruption and gut dysbiosis—offer a plausible, if incompletely verified, explanation for this pattern. Cytokine-driven inflammation is already pronounced in early AP (Li et al., 2020); AP-associated systemic inflammatory responses may compromise mucosal barrier integrity and function, promoting the overgrowth of opportunistic organisms and facilitating their translocation to peripancreatic necrotic tissue, potentially constituting a critical pathological substrate for IPN development (Wu et al., 2023; Ahuja et al., 2017). Zhu et al. demonstrated that gut microbiota perturbation and bacterial invasion of epithelial cells occur even in mild AP (Zhu et al., 2019), indicating that mucosal barrier injury is initiated at a very early disease stage (Wang et al., 2023). Regarding dysbiosis, Liu et al., drawing on fecal samples collected within three days of admission, documented early overgrowth of opportunistic pathogens alongside marked depletion of short-chain fatty acid-producing bacteria in AP patients (Liu et al., 2024). The convergence of these two processes may be consistent with a clinical context favoring enteric translocation: Ammori provided a classic description of how a disrupted intestinal barrier permits luminal organisms to seed peripancreatic necrotic tissue (Ammori, 2003); Glaubitz et al. subsequently demonstrated that Treg cell-mediated duodenal barrier injury can directly drive bacterial translocation to necrotic areas in severe AP (Glaubitz et al., 2023); and animal experimental data further suggest that gut microbial colonization causally exacerbates pancreatic pathological injury (Liu et al., 2024). If these mechanisms are operative early in the disease course—before organ failure has supervened—conditions potentially associated with enteric translocation may already be present, which may partly account for why Non-POF patients already harbor an ICU-associated peripancreatic microbiota closely resembling that of the POF group. This remains an inference, however, and direct prospective evidence is needed before it can be accepted as established.

The absence of a detectable organ failure effect on pus microbiota may seem inconsistent with reports from the critical care literature linking the degree of organ dysfunction to gut dysbiosis in sepsis (Liu et al., 2026; Park et al., 2024). The distinction, however, is ecologically fundamental: those studies examined the intestinal microbiome, whereas the present work characterizes an established local infectious focus in the abdominal cavity—two compartments shaped by different forces. Once ICU-associated pathogens colonize peripancreatic necrotic tissue, the local microecology may reach a degree of compositional stability under physical containment and intense antibiotic selection pressure, which may partly explain the limited differences observed between POF and Non-POF groups at the time of sampling; whether this holds in practice requires direct evidence. From a clinical perspective, the timing of targeted antimicrobial initiation has been consistently linked to outcomes once IPN is established, with delayed coverage associated with higher complication rates and mortality (Mayerle et al., 2005; Büchler et al., 2000); current pancreatitis guidelines similarly recommend early antibiotic therapy when infected necrosis is suspected on clinical or imaging grounds (IAP/APA/EPC/IPC/JPS Working Group, 2025). The microbiological pattern described here suggests that prospective evaluation of antimicrobial coverage breadth at the point of IPN diagnosis may be warranted, a hypothesis requiring validation before clinical implementation.

It is notable that pre-sampling antibiotic duration was substantially longer in both AP subgroups than in the Non-AP group, a difference closely intertwined with the characteristically prolonged disease course and extended ICU management of severe acute pancreatitis (IAP/APA/EPC/IPC/JPS Working Group, 2025). Prolonged broad-spectrum antibiotic exposure, extended ICU stay, and repeated invasive procedures collectively constitute the typical clinical context of severe AP and cannot be readily disentangled from the disease process itself. Within this context, gut microbiota alterations, mucosal barrier disruption, and antibiotic selective pressure interact to shape the microbial composition of peripancreatic pus. A further internal observation of note is that, within the AP population, the Non-POF group had longer pre-sampling antibiotic exposure than the POF group, yet the pus microbial community composition of the two subgroups was highly similar. This observation supports interpreting these findings within the broader clinical context of severe AP. The microbial profile described in this study therefore reflects the infectious microecology of severe AP patients within their real-world clinical setting, with residual confounding by antimicrobial exposure and other ICU-related factors remaining possible.

The differential enrichment of specific commensal species in non-pancreatitis pus carries its own interpretive value. *E. faecalis* remained inversely associated with AP status after separate adjustment for comorbidity burden, disease severity, pre-sampling antibiotic exposure, and ICU admission-to-sampling interval. This consistency supports the robustness of the observed association after accounting for these key available clinical factors. In contrast, the inverse associations observed for *S. hominis*, *M. osloensis*, and *A. johnsonii* remained significant after adjustment for CCI, APACHE II score, or ICU admission-to-sampling interval, but were attenuated after adjustment for pre-sampling antibiotic duration. This pattern suggests that cumulative antimicrobial exposure may partly contribute to the distribution of these taxa. Rather than representing established diagnostic markers, these findings support the distinct microbial ecology of Non-AP intra-abdominal infection observed in this cohort. *E. faecalis* is a numerically dominant intestinal commensal; Wang et al. documented detection rates of 30.8% in lower gastrointestinal and 18.2% in upper gastrointestinal perforation cases by peritoneal culture (Wang et al., 2020), and its relative scarcity in pancreatitis pus supports differences in microbial composition and likely infectious context between the two infection types. *S. hominis*, a predominant skin colonizer, has been reported to gain access to the peritoneal cavity via catheter-associated cutaneous contamination (Monsen et al., 2000), suggesting that skin-derived organisms contribute to the microbial composition of perforation-associated abdominal infection. *M. osloensis* is a normal upper respiratory tract commensal (Shin et al., 2026); its peritoneal detection most likely reflects environmental exposure related to the drainage procedure, though this interpretation awaits further verification.

The random forest models provided a quantitative assessment consistent with the community-level findings. AUC values for the pancreatitis identification task (AP vs. Non-AP) exceeded those of the three-group classification at every taxonomic level examined (0.769–0.843 vs. 0.587–0.731), a consistent performance gap that mirrors the biological reality of near-identical community structures in the POF and Non-POF groups—a similarity that inherently limits the discriminatory capacity of three-group models. The family-level pancreatitis identification model achieved the highest AUC (0.843), a performance broadly comparable to machine learning models built on gut microbiome data for gastrointestinal disease classification: Hu et al. reported AUCs of 0.818–0.864 for an IBD-NAFLD comorbidity identification model integrating multiple algorithms (Hu et al., 2025), and a systematic review of 45 studies confirmed that gut microbiome-based colorectal cancer diagnostic models routinely exceed 80% AUC (Upadhyay et al., 2025), suggesting that pus microbiota carry disease-discriminatory information of comparable density to intestinal community features. The top-ranked family-level features—Oxalobacteraceae, Eubacteriales Family XIII Incertae Sedis, and Propionibacteriaceae—are all gut- or environment-derived commensal families, fully consistent with the differential species analysis, and the convergence of two independent analytical approaches on the same biological signal strengthens the central argument for infection-source-associated microbial patterning. It should be noted that the random forest models were constructed and evaluated within the same cohort; the AUC values reported accordingly reflect within-sample discriminatory capacity rather than prospectively validated predictive performance, and external validation in independent cohorts will be required before any clinical application can be considered.

This study has several limitations. First, the single-center retrospective design and relatively limited sample size may restrict generalizability across institutions with different ICU pathogen ecology, antimicrobial practices, and resistance patterns. Second, the substantially longer pre-sampling antibiotic exposure in the AP groups represents an important potential source of residual confounding; because antimicrobial exposure is closely intertwined with the prolonged disease course and ICU management of severe AP, its independent contribution cannot be fully disentangled. Third, ARG profiling was based primarily on mNGS rather than systematic phenotypic susceptibility testing, and paired genotype–phenotype data were available only for a subset of cases. Fourth, the Non-AP comparator was etiologically heterogeneous, and only 13 Non-AP patients had organ failure, limiting the power of organ-function-matched analyses and potentially affecting some between-group comparisons. Fifth, although drainage modalities and the ICU admission-to-sampling interval were comparable across groups, residual procedure-related variation cannot be completely excluded. Finally, the cross-sectional design precludes assessment of longitudinal microbial dynamics, and the internally validated random-forest models require external validation before any clinical application.

## 5 Conclusion

Metagenomic sequencing of 115 intra-abdominal pus samples from a surgical ICU yielded three principal findings. Compared with non-pancreatitis infection, AP-associated pus exhibited a distinct microbial community characterized by reduced diversity and predominance of ICU-associated pathogens with broad ARG detection profiles, including frequent detection of carbapenemase-related and glycopeptide resistance genes. This community pattern was similar in AP patients with and without POF, supporting its description as an AP-associated microbial pattern. Together, these microbiome and resistance-gene findings may complement resistance-risk assessment and empirical antimicrobial selection in critically ill patients with suspected IPN when interpreted alongside culture results, phenotypic susceptibility testing, and individual clinical factors. Prospective multicenter validation is warranted.

## Data Availability

The datasets presented in this study can be found in online repositories. The names of the repository/repositories and accession number(s) can be found at: https://ngdc.cncb.ac.cn/gsa, CRA046720.

## Footnotes

1
ftp://ftp.ncbi.nlm.nih.gov/genomes/genbank/
