# Supplementary material for: Metagenomic landscape of peripancreatic necrotic pus in acute pancreatitis: pathogen ecology, resistance gene profiles, and disease-associated microbial patterns
Source: Front Microbiol. 2026 Sep 10;17:1950013. doi: 10.3389/fmicb.2026.1950013 (PMC13601262; doi:10.3389/fmicb.2026.1950013)
Supplement: Supplementary file 1 [file Table_1.docx]

Supplementary Material

**Supplementary Figure 1.** Detection rates of beta-lactam resistance gene subcategories (Carbapenemase, ESBL, AmpC, and Penicillinase/Others) across the three groups. POF, persistent organ failure; AP, acute pancreatitis. **P*<0.05, ***P*<0.01; ns, not significant.

**Supplementary Figure 2.** Box plots of pairwise Bray-Curtis dissimilarity of pus microbiota between organ-function-matched groups. The Non-AP group was stratified by organ function status at sampling into a subgroup with organ failure (Non-AP-OF, n=13) and a subgroup without organ failure (Non-AP-NOF, n=18), compared with the POF and Non-POF groups, respectively. POF, persistent organ failure; AP, acute pancreatitis.

**Supplementary Figure 3.** Sensitivity analysis of microbial diversity after restricting the Non-AP group to gastrointestinal perforation-associated or postoperative gastrointestinal surgery-associated infection (n = 23). (A) PCA scatter plot of microbial community composition. (B) PCoA scatter plot based on Bray-Curtis dissimilarity. (C) Pairwise Bray-Curtis dissimilarity among the three groups. (D-G) Comparisons of Shannon, Simpson, ACE, and Chao1 indices. *P* values indicate pairwise comparisons. POF, persistent organ failure; AP, acute pancreatitis.

**Supplementary Figure 4.**　Genus-level microbial community composition of pus samples across the three groups. (A) Stacked bar chart of top 20 genera by mean relative abundance across groups; “other” denotes the cumulative abundance of genera beyond the top 20. (B) Venn diagram of shared and group-exclusive genera across the three groups.

Supplementary Table S1. Culture-based carbapenem susceptibility results and mNGS carbapenemase-related gene detection in 18 culture-positive *Klebsiella pneumoniae* cases.

| **Case ID** | **Group** | ***K. pneumoniae* by mNGS** | **Paired analysis** | **Carbapenemase gene by mNGS** | **Carbapenem phenotype** |
| --- | --- | --- | --- | --- | --- |
| Case 01 | POF | Detected (1,237,844) | Yes | KPC-2 | CRKP |
| Case 02 | POF | Detected (1,686,497) | Yes | KPC | CRKP |
| Case 03 | POF | Detected (16,680) | Yes | Not detected | Carbapenem-susceptible |
| Case 04 | POF | Detected (49,970) | Yes | KPC-2 | CRKP |
| Case 05 | POF | Detected (396,792) | Yes | KPC | CRKP |
| Case 06 | POF | Detected (114,799) | Yes | KPC-24 | CRKP |
| Case 07 | POF | Detected (1,343,256) | Yes | NDM-1 | CRKP |
| Case 08 | POF | Detected (2,792,714) | Yes | Not detected | Carbapenem-susceptible |
| Case 09 | POF | Detected (1,126,299) | Yes | KPC-2 | CRKP |
| Case 10 | Non-POF | Detected (7,796) | Yes | KPC-3 | CRKP |
| Case 11 | Non-POF | Detected (465,862) | Yes | Not detected | CRKP |
| Case 12 | Non-POF | Detected (110,784) | Yes | KPC | CRKP |
| Case 13 | Non-AP | Detected (194,265) | Yes | Not detected | CRKP |
| Case 14 | Non-AP | Detected (539) | Yes | Not detected | Carbapenem-susceptible |
| Case 15 | POF | Not detected | No | Not detected | CRKP |
| Case 16 | Non-POF | Not detected | No | Not detected | CRKP |
| Case 17 | Non-AP | Not detected | No | Not detected | Carbapenem-susceptible |
| Case 18 | Non-POF | Not detected | No | Not detected | CRKP |

K. *pneumoniae* by mNGS indicates mNGS detection of *K. pneumoniae*; parentheses indicate species-specific read counts. Carbapenemase gene by mNGS indicates carbapenemase-related ARGs detected by mNGS. Carbapenem phenotype was determined by culture-based susceptibility testing. Paired analysis included cases with *K. pneumoniae* detected by both methods and matched specimen type and sampling time. POF, persistent organ failure; AP, acute pancreatitis; CRKP, carbapenem-resistant *K. pneumoniae*.

Supplementary Table S2. Pairwise comparisons of alpha-diversity indices among the three patient groups.

| **Index** | **Comparison** | **Unadjusted P** | **Bonferroni-adjusted P** | **Rank-biserial correlation** | **95% CI** |
| --- | --- | --- | --- | --- | --- |
| Shannon | POF vs Non-POF | 0.563056 | 1.000000 | 0.076 | -0.173 to 0.335 |
| Shannon | POF vs Non-AP | 0.000533 | 0.001599 | -0.491 | -0.733 to -0.225 |
| Shannon | Non-POF vs Non-AP | 3.10×10^-5^ | 9.30×10^-5^ | -0.549 | -0.759 to -0.319 |
| Simpson | POF vs Non-POF | 0.472468 | 1.000000 | 0.094 | -0.161 to 0.339 |
| Simpson | POF vs Non-AP | 0.001705 | 0.005115 | -0.448 | -0.682 to -0.187 |
| Simpson | Non-POF vs Non-AP | 4.90×10^-5^ | 0.000147 | -0.536 | -0.750 to -0.295 |
| ACE | POF vs Non-POF | 0.269443 | 0.808329 | -0.157 | -0.447 to 0.134 |
| ACE | POF vs Non-AP | 0.002090 | 0.006270 | -0.460 | -0.715 to -0.186 |
| ACE | Non-POF vs Non-AP | 0.001102 | 0.003306 | -0.449 | -0.689 to -0.184 |
| Chao1 | POF vs Non-POF | 0.250721 | 0.752163 | -0.150 | -0.399 to 0.107 |
| Chao1 | POF vs Non-AP | 0.000229 | 0.000687 | -0.534 | -0.756 to -0.290 |
| Chao1 | Non-POF vs Non-AP | 0.000353 | 0.001059 | -0.486 | -0.712 to -0.238 |

Pairwise comparisons were performed using two-sided Wilcoxon rank-sum tests. For each alpha-diversity index, *P* values from the three pairwise comparisons were adjusted using the Bonferroni methods. Rank-biserial correlation was calculated as the effect size, with 95% confidence intervals estimated using 5,000 bootstrap resamples. Positive effect sizes indicate higher values in the first-listed group, whereas negative values indicate higher values in the second-listed group. POF, persistent organ failure; AP, acute pancreatitis; CI, confidence interval.
